# Is physician online information sharing always beneficial to patient education? An attention perspective

**DOI:** 10.3389/fpubh.2022.987766

**Published:** 2022-08-30

**Authors:** Feng Guo, Apan Zhou, Wenhao Chang, Xinru Sun, Bo Zou

**Affiliations:** ^1^College of Management and Economics, Tianjin University, Tianjin, China; ^2^School of Business, Sun Yat-sen University, Guangzhou, China

**Keywords:** online information sharing, patient education, attention theory, online health platforms, online reputation, offline expertise

## Abstract

**Aims:**

With the development of information technology, online health platforms and physician online information sharing play an important role in public health management and patient education. Is physician online information sharing always beneficial to patient education? From the attention perspective, this study aims to explore how physician online information sharing influences patient education, considering the contingent roles of physician online reputation and offline expertise.

**Methods:**

A 6-month panel data of 61,566 physician-month observations from an online health platform in China was used to tested the proposed hypotheses. Considering the inefficiency and estimated bias of the ordinary least squares regression model, this study conducted the fixed models to test the direct and moderating effects.

**Results:**

The results indicate that physician online information sharing is positively related to potential patient education, while the relationship between physician online information sharing and realized patient education is an inverted U-shape. Physician online reputation enhances the positive relationship between physician online information sharing and potential patient education, but physician offline expertise weakens the abovementioned relationship. In addition, physician offline expertise flattens the curvilinear effect of physician online information sharing on realized patient education.

**Conclusion:**

This study contributes to the literature about attention theory and information sharing for patient education, and provides implications for practice.

## Introduction

With the advancement of online technologies, online health platforms (OHPs) have become one of the most convenient channels for patients to obtain health-related information and for physicians to spread their knowledge, experiences, and skills (1–3). On some OHPs, physicians impart their knowledge to patients by publishing health articles (4), and patients browse pages, search for useful information, and read health articles to improve their health behaviors and status (5). Physicians' information sharing on OHPs transcends temporal and geographic restrictions, and thus helps mitigate the unbalanced distribution of medical resources and promote patient outcomes (5–7). Existing research has examined the outcomes of physicians' information sharing on OHPs. Bryant et al. (8) found that information sharing improves physician–patient relationships and health service quality; Meng et al. (5) indicated that information sharing can increase physicians' online revenue by attracting paid consulting. In fact, patients are target receivers of physicians' shared information, playing a crucial role in achieving the beneficial outcomes of information sharing (5). In this vein, one of the most important outcomes of physicians' information sharing is patient education.

Patient education refers to the activities designed to improve patients' health behaviors and health status (9). It is distinguished as potential patient education through patients' visiting and realized patient education by patients' reading. With the prevalence of OHPs, physicians' online information sharing has become an available way to implement patient education. However, the constantly increasing volumes of health information cause processing problems for patients seeking relevant knowledge and give rise to competition for limited attention (10, 11). As a selective mechanism to allocate cognitive resources (12, 13), limited attention influences individuals' information behaviors significantly (11). For instance, when searching for medical guidance on OHPs, a patient might only pay attention to some of the articles presented on the first few webpages because the patient does not have enough time or cognitive resources to focus on all results. In spite of the richness of attention research (14, 15), the literature does not provide sufficient insights in explaining the relationship between information sharing and patient education on OHPs from the attention perspective. For a better understanding of this issue, limited attention as a fact should be taken into account.

Actually, the information decision-making process is a trade-off between the benefits and the costs of limited attention (14), and such behaviors are not independent of the context. On OHPs, online reputation is previous patients' evaluations of physicians' performance (16). In comparison, expertise is indicated by the clinic title, which is obtained after years of clinical work and professional assessment (17). Both online reputation and offline expertise are available signals about a physician's experience and competence, and might modify patients' perceived value of health information shared by the physician (1, 18). Therefore, online reputation and offline expertise may reshape the trade-off relationship between benefits and costs of attention, playing a contingent role in the process where physician online information affects potential and realized patient education. To obtain a better understanding of information sharing and patient education on OHPs, this study aims to explore the following research questions:

*How does physician online information sharing influence potential patient education and realized patient education?*
*Are the above relationships moderated by online reputation and offline expertise?*

According to attention theory, attention functions as an information filter for human beings to allocate limited perceptual and cognitive resources (12, 19). Commonly, attention processes can be either stimulus-driven or goal-driven (20–22). As a more stimulus-driven process, patients' visiting is expected to increase with an increase of physician information sharing since patients' attention could be captured by topics of interest in health articles (12, 23). However, patients' reading is a more goal-driven process where information overload dampens patients' attention to physicians' articles (24). Hence, the relationship between physician online information sharing and realized patient education is expected to be an inverted U-shape pattern. Online reputation acts as a trustable signal to increase patients' perceived information value (15, 25) and anticipated benefits from reading health articles (26, 27). Therefore, online reputation may strengthen the relationship between information sharing and potential patient education, and steepen the inverted U-shape relationship between online information sharing and realized patient education. Offline expertise is obtained after years of clinical work and professional assessment, and may amplify the worth of information and heighten the expected attention cost of reading health articles (17, 28, 29). Therefore, offline expertise may weaken both the relationships between information sharing and patient education mentioned above.

For testing of our hypotheses, a 6-month panel data of 61,566 physician-month observations from an OHP in China are collected. The results provide empirical support for most of our hypotheses, with an only exception that the moderating role of online reputation in the relationship between physicians' online information sharing and realized patient education was not supported. To be more specific, physician online information sharing positively affects potential patient education, while having an effect on realized patient education in an inverted U-shape pattern. Physician online reputation promotes the positive relationship between physician information sharing and potential patient education, but physician offline expertise weakens the abovementioned relationship. Moreover, physician offline expertise flattens the curvilinear relationship between physician online information sharing and realized patient education.

This study also makes several contributions to the literature. First, this study contributes to the attention literature by introducing the attention theory to track the mechanism of physician online knowledge sharing and patient education. By applying an attention perspective, this study clarified how physician online knowledge sharing influences potential and realized patient education in different patterns. Second, this study contributes to the online reputation and patient education literature by uncovering the contingent effect of online reputation in the process of patient education. The accomplishment of patient education by physicians' information sharing is actually a decision-making process for patients, which depends on the context (14, 30). Our study clarifies how physicians' online reputation reshapes patients' decision-making on acquiring knowledge from physicians. Finally, this study contributes to the extant offline expertise and patient education literature by revealing the contingent effect of offline expertise in the process of patient education. In spite of the recent focus on physicians' expertise (5, 31), more insights on its role in patient education are needed. Therefore, this study sheds light on the moderating role of physicians' offline expertise on the trade-off between the benefits and the costs of limited attention to online health information, complementing the understanding of the effect of expertise.

The article is structured as follows: after the introduction, theory background and hypotheses are proposed in Section Theory background and hypotheses. Section Methodology illustrates the method and Section Results presents results. Moreover, findings, contributions, implications, limitations and conclusion are detailed in Section Discussion.

## Theory background and hypotheses

### Attention theory

Early research on the essence of consciousness and volition highlights the importance of the concept of attention (22). According to previous attention literature, attention has been defined as the allocation of limited cognitive processing capacity toward selective concentration on particular information (32, 33). Limited attention implies information cost and constrained choices (34). In fact, attention functions as an information filter, selecting some information for further processing while inhibiting others from being processed (19, 35). The mechanism of attention is the most efficient way for human beings to allocate limited perceptual and cognitive resources, enabling information receivers to become active seekers and processors as well (12). Commonly, attention processes can be either stimulus-driven or goal-driven (20–22). The former refers to the case when one's attention is captured by some external event, while the latter happens when one's attention is controlled voluntarily for a certain goal (12).

Attention theory is well-established with an expansion of research examining the mechanism of attention and identifying it as an important driver of various outcomes over the past few decades (36). These works identified the fundamental principle that attention is a limited cognitive resource (37, 38), and individuals selectively ignore some of the information that competes for their attention (36, 39). There is also a wide range of management researchers focusing on various types of attention, including consumer attention, investor attention, employee attention, user attention, and regulatory attention (14, 24, 36). Moreover, with the prevalence of online platforms, several recent studies try to enhance the understanding of user behaviors and information networks from the perspective of attention (24, 40).

The widespread information explosion now-a-days on online platforms, including OHPs, leads to competition for limited attention, which has a significant effect on individuals' information decisions and behaviors (11). On OHPs, physicians publish online articles for the purposes of health promotion and patient education, while patients seek relevant and useful information such as medical knowledge and professional advice (5, 41). Physicians' information sharing benefits both patients and physicians themselves, promoting the prosperity of the OHPs (5, 42). However, given the ever-increasing shared information on OHPs causes difficulties in accessing and absorbing knowledge, which may affect the achievement of public health management and patient education (11). An attention perspective is therefore needed and vital to enhance the understanding of physician information sharing and possible patient education.

The attention theory helps uncover the mechanism through which patients decide whether to allocate or not their limited cognitive resources to health articles shared by physicians (12). It thus offers a visualized framework to enhance the understanding of how physician online information sharing provokes potential patient education by patients' visiting and realized patient education by patients' reading. According to attention theory, patients' visiting is similar to a stimulus-driven attention process, and the information sharing by physicians may initiate potential patient education as patients' attention could be captured by contents of interest in shared health articles (12, 23). However, patients' reading is a goal-driven process where the patients' information decision is the trade-off between the benefits and the costs of attention, and information overload dampens patients' attention to physicians' articles (24). Moreover, online reputation and professional expertise, as easily accessible signals on OHPs for physicians' experience and competence, may reshape patients' perceived value of health articles, and thus affect their information decisions (16, 43). Therefore, it is necessary to consider the contingent role of physicians' online reputation and offline expertise in the process of physician online information sharing and patient education.

### Online information sharing and patient education

Online information sharing refers to physicians' health and medical information sharing that is available for patients on OHPs (44). Patient education refers to the activities designed to improve patients' health behaviors and health status (9), and is distinguished as potential patient education through patients' visiting and realized patient education by patients' reading. Patients' visiting is defined as the total number of a physician's homepage visits by patients on the OHP (45). Patients' reading is defined as the amount of reading of a physician's shared health articles by patients (5). Within OHPs, shared health content is so extensive that patients have access to an almost limitless selection of information, which competes for their limited time and cognitive resources (36). However, limited attention implies that patients' visiting is caught only by a certain subset of the available articles, and their further reading happens only when the perceived benefits exceed the costs of attention (14, 36).

Physicians' online information sharing positively influences potential patient education. The more health articles a physician has on an OHP, the more likely their articles are to be included in patients' subset of alternatives identified by limited attention from the full spectrum of alternative information during browsing the webpage (36, 46). Further, with the increase of articles published, more health topics are covered, leading to a higher possibility of addressing patients' information needs and filling their specific knowledge gaps (47). In other words, the more the physician's health articles are shared, the more likely a valuable article exists to catch patients' attention and trigger their visiting. On the basis of the above argument, we propose the following hypothesis:

**H1:** *Physicians' online information sharing is positively related to potential patient education*.

Physicians' online information sharing is associated with realized patient education in a non-linear pattern, and this is because patients' decision to read the article on the OHP or not depends on the trade-off between the benefits and the costs of attention (14). As reading articles on the benefits from reading a shared health article outweigh the cost (48). The benefits of attention to the shared articles include gains such as learning health knowledge and addressing health problems, while the costs could be attributed to the information-processing time, cognitive resources, and opportunity costs (14).

As the number of shared articles by a physician gradually increases from low to moderate, the value of the shared information perceived by patients increases. For instance, the articles may discuss the patients' specific health issues, fill their relevant knowledge gaps, and help to improve their health behaviors. There is also a growing cost for the attention to select an article from all the alternatives and then read it as the volume of information increases (36). However, at this stage, the attention is at a relatively low level because the quantity of information is under the threshold of personal cognitive capacity and information overload has not yet occurred (24, 49). Therefore, physicians' online information sharing positively affects realized patient education as the perceived benefits of attention outweigh the cost.

However, when the number of shared articles is greater than a certain threshold, the cost of attention becomes more conspicuous. In essence, the attention allocated to a health article comes at the expense of attention allocated to all other articles in the patient's subset of alternatives (36, 50). As a result, the opportunity cost of attention increases with an increasing quantity of information available (51). Moreover, information overload occurs when the number of shared articles exceeds what a patient can deal with (24, 49). Information overload causes a sense of fatigue, hampers the capability to process information, and distorts the real value of information, thus downplaying the patient's original interest in the health articles (52). When the quantity of shared articles exceeds a certain threshold, the perceived cost of attention is higher than the benefit. Therefore, in this phase, physicians' online information sharing negatively affects realized patient education. From the above arguments, we propose the following hypothesis:

**H2:** *There is an inverted U-shape relationship between physicians' online information sharing and realized patient education*.

### The moderating effect of online reputation

Online reputation is defined as patients' evaluations of physicians' performance, reflecting their capability and popularity (16). Reputation plays a signaling role to reduce patients' concerns about quality risk and uncertainty on OHPs where there is severe health information asymmetry between physicians and patients (5). Online reputation is a trustable signal because it is an extrinsic cue about the social approval of physicians' competence (18, 53). Moreover, reputation represents a valuable and rare resource, and is thus a common source of competitive advantage (25, 53). Online reputation might modify patients' perception of information value and have a contingent effect on their information decisions.

Online reputation positively moderates the relationship between physicians' online information sharing and potential patient education. First, as a valuable and rare resource, a good online reputation makes the information shared by the physician more interesting and attractive to patients (53, 54). In this case, physicians' shared articles are more likely to attract patients' attention and induce their visiting. Second, a good reputation is a reliable signal about high quality and value of health articles shared by the physician (18, 43). In this condition, with their attention caught by health articles, patients are more inclined to believe that their health issues could be addressed, and are thus more willing to visit the physician's homepage to obtain more information. On the basis of the above arguments, we propose the following hypothesis:

**H3:** *Online reputation strengthens the positive relationship between physicians' online information sharing and potential patient education*.

Online reputation also moderates the inverted U-shape relationship between physicians' online information sharing and realized patient education. As discussed before, reading articles on an OHP is a more goal-driven process, which is implemented only when expected profits can be realized (14, 48). When the total number of articles shared is under a certain threshold, online reputation acts as both a source of competitive advantage and a signal about expertise and capacity, increasing perceived benefits of attention to the information shared by the physician (18, 25, 53). Under this circumstance, the trade-off between benefits and costs of attention inclines to the side of benefits, and patients are more willing to read shared health articles. As a result, online reputation strengthens the positive relationship between physicians' online information sharing and realized patient education.

However, good online reputation may also be regarded as a means to attract patients and consultations, and increases patients' expectations of the value and benefits of reading shared information, especially when there is a large amount of shared articles (10, 26). If patients cannot obtain the anticipated profits from reading the health articles, it is hard for them to keep their attention focused there (14). In this case, a good reputation magnifies the difficulties for the physician's shared information to meet patients' expectations and gain their attention. As a consequence, online reputation strengthens the negative relationship between physicians' online information sharing and realized patient education when the volume of shared information is relatively high. From the above arguments, we propose the following hypothesis:

**H4:** *Online reputation steepens the inverted U-shape relationship between physicians' online information sharing and realized patient education*.

### The moderating effect of offline expertise

Offline expertise refers to the experience of continuous health service and medical capability, and is indicated by the clinic title, which is classified into four hierarchical levels (1, 17). The clinic title is obtained after a long period of clinical work and professional assessment. It is an authoritative signal of physicians' experience and competence, which is not affected by their online activities (1, 29). Generally, physicians with a high clinic title have high incomes from hospitals and are more respected and trusted by patients (17). Offline expertise is expected to reshape patients' perception of information from physicians, and thus play a contingent role in their information decisions.

Offline expertise weakens the positive relationship between physicians' online information sharing and potential patient education. Physicians with higher clinic titles are regarded as experts in their medical field, and their articles are perceived as more rewarding to patients (55, 56). Valuable information receives attentional priority, and once the shared information enters patients' browsing range, it draws the patients' attention (23). Further, physicians with higher professional titles are more likely to obtain active goal-driven attention from patients because patients tend to choose medical services from higher-titled physicians, and are thus inclined to seek health knowledge shared by them as well (1, 29). Therefore, for those physicians who have higher clinic titles, the growth of their page visits is less dependent on the total amount of articles. On the basis of the above arguments, we propose the following hypothesis:

**H5:** *Offline expertise weakens the positive relationship between physicians' online information sharing and potential patient education*.

Offline expertise also moderates the inverted U-shape relationship between physicians' online information sharing and realized patient education. To read or not depends on the trade-off between benefit and cost of attention (14). When the total quantity of articles shared is relatively small, offline expertise weakens the positive relationship between physicians' online information sharing and realized patient education. This is because information sharing from experts (physicians with high professional titles) is considered more valuable guidance (17). Patients tend to believe that they will benefit from reading experts' shared health articles even if few topics are covered and they are not immediately relevant. Thus, for physicians with higher professional expertise, the benefits of attention will not be hampered so much as the number of shared articles decreases.

When the total number of articles shared is relatively high, offline expertise alleviates the negative relationship between physicians' online information sharing and realized patient education. First, professional title is an authoritative signal of physicians' experience and competence (1, 29). There is higher perceived benefit from attention to information shared by physicians with higher professional titles. Second, patients hope to obtain medical service from experts, but experts are essentially scarce in the medical and health market, especially when the disequilibrium of medical resources impedes the accessibility to expert treatment (28, 29). The scarcity of experts heightens the expected attention cost patients are willing to pay. In this instance, patients gain perceived profits from reading health articles, and are thus more likely to read them even when there is a large amount of shared information. On the basis of the above arguments, we propose the following hypothesis:

**H6:** *Offline expertise flattens the inverted U-shape relationship between physicians' online information sharing and realized patient education*.

**Figure 1:**
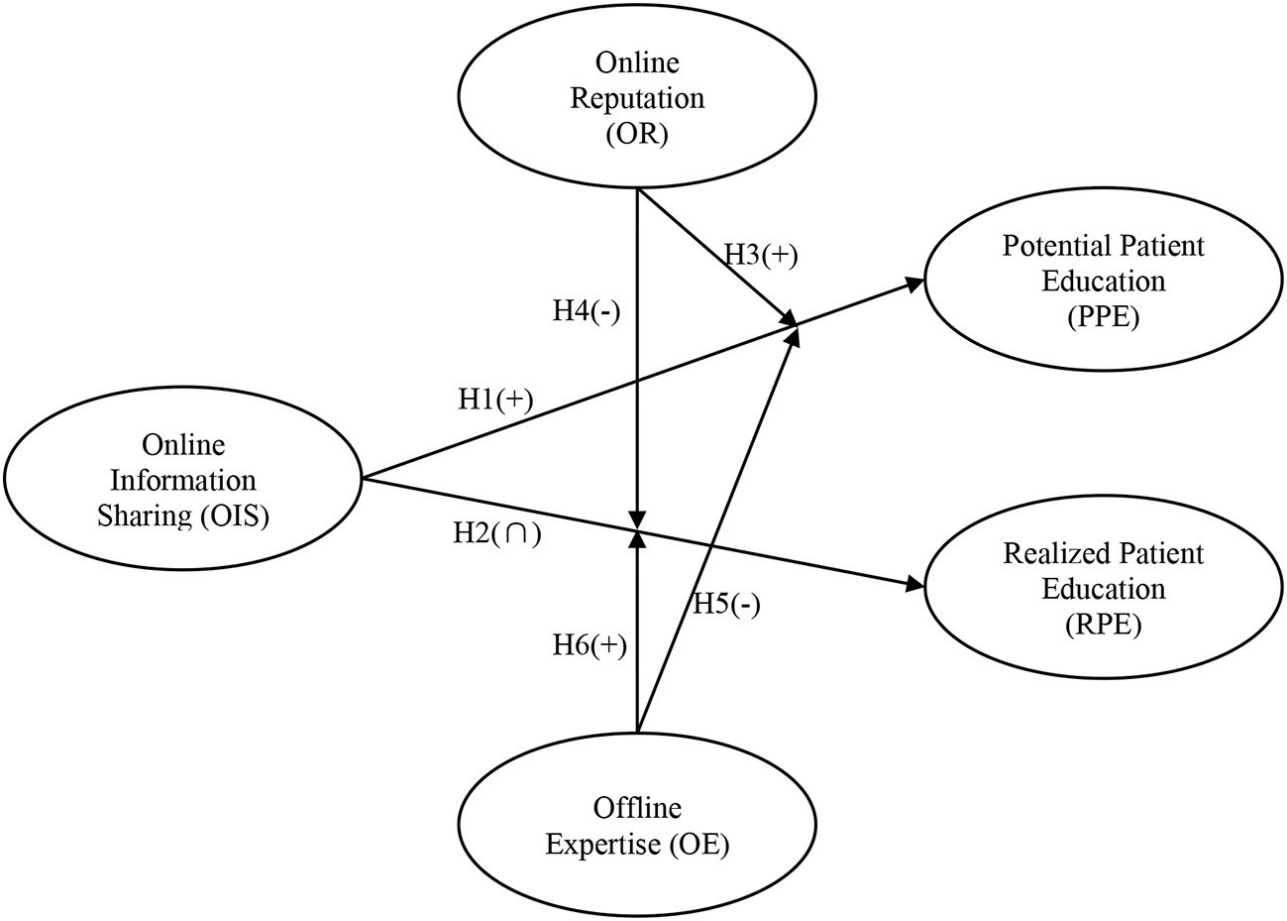
Research model.

In summary, the research model is presented in Figure 1.

## Methodology

### Data collection

We choose the OHP “haodf.com” as our data source because the data collected from this online platform are objective and avoid self-reporting bias effectively (5). Additionally, the database haodf.com provides us with the following advantages. First, the platform has a large number of users and physician–patient interactions, which enables us to obtain abundant data for the study. It is reported that this platform brings together more than 200,000 physicians from different hospitals across the whole country and offers service to more than 58,000,000 patients online (5). Second, this online platform makes it possible for us to explore physicians' online information sharing and patient education. We focused on public information sharing and patient education for research purposes. The free and public characteristics of the published articles on the online platform make it convenient for patients to access and absorb; hence, patient education can be identified and achieved.

Using a Java-based web crawler, we successfully collected the article publications and website data statistics of 66,563 physicians over 6 months (February 2017 to July 2017). After deleting some incomplete data, we finally obtained an unbalanced panel of 19,022 physicians with 61,566 physician-month observations.

### Measures

#### Dependent variables

Patient education refers to the activities designed to improve patients' health behaviors and health status (9), and is segmented into potential patient education through patients' visiting and realized patient education by patients' reading. We used the number of patients visiting physicians' homepages to measure potential patient education. As for realized patient education, we measured it by the number of article readings of physicians on the OHP.

#### Independent variables

Online information sharing is defined as physicians' health and medical information sharing that is available for patients on the OHP (44). From previous studies (5, 31), we chose the number of free health-related articles shared by physicians on the online platform as the measure of this variable. Online reputation refers to patients' evaluations of physicians' performance, reflecting their capability and popularity (16). We used the number of patients received by physicians to measure online reputation. Offline expertise refers to the experience of continuous health service and medical capability (1, 17). It was measured by the offline titles of physicians, from the lowest to the highest rankings; the offline titles in China are resident physician, attending physician, associate chief physician, and chief physician (5). In this study, we ranked this variable from 1 to 4 to represent the physicians' offline title.

#### Control variables

Following previous studies (5, 31), we added several variables as controls. First, Online time refers to the length of time the physicians had been using the OHP. We used the time in months that each physician had been using the OHP for measuring this variable. Second, the number of online gifts and votes that physicians receive may influence patient education. Therefore, we controlled Gift and Vote variables for more realistic results. Finally, the number of online thank-you letters from patients was used to measure the last control variable—Thank-you.

Considering the magnitude of the original data, according to Kafouros et al. (57), we used the logarithm of all variables except offline expertise. Table 1 lists an overview of all the variables in this study.

**Table 1:** The overview of all variables.

**Variables**	**Description**	**Mean**	**SD**	**Min**	**Max**
Potential patient education	The number of patients visiting a physicians' homepage	11.571	1.754	5.024	17.719
Realized patient education	The number of article readings of physicians	7.777	1.170	1.099	12.891
Online information sharing	The number of free health-related articles shared by physicians	1.745	1.263	0.000	7.550
Online reputation	The number of patients received by physicians	4.584	2.116	0.000	10.672
Offline expertise	The offline tittles of physicians	2.986	0.908	1	4
Online time	The opening time of physicians	7.221	0.817	2.303	8.030
Gift	The number of online gifts from patients	1.744	1.599	0.000	7.920
Vote	The number of votes physicians received	2.195	1.321	0.000	7.046
Thank-you	The number of online thank you letters from patients	1.181	1.187	0.000	6.084

### Data analysis

In testing our hypotheses, we introduced the following equations to estimate the effects of online information sharing (*OIS*) on potential patient education (*PPE*) and realized patient education (*RPE*):


$$\begin{array}{l} PPE_{it}=\beta_{0}+\beta_{1}Onlinetime_{it}+\beta_{2}Gift_{it}+\beta_{3}Vote_{it}\\ \;+\beta_{4}Thank-you_{it}+\beta_{5}OIS_{it}+\beta_{6}OIS_{it}^{2}+\beta_{7}OR_{it}\\ \;+\beta_{8}OR_{it}\times \beta_{9}OIS_{it}+\beta_{10}OR_{it}\times \beta_{11}OIS_{it}^{2}\\ \;+\beta_{12}OE_{it}+\beta_{13}OE_{it}\times \beta_{14}OIS_{it}+\beta_{15}OE_{it}\\ \;\times \beta_{16}OIS_{it}^{2}+\mu_{it}\\ RPE_{i,t}=\beta_{0}+\beta_{1}Onlinetime_{it}+\beta_{2}Gift_{it}+\beta_{3}Vote_{it}\\ \;+\beta_{4}Thank-you_{it}+\beta_{5}OIS_{it}+\beta_{6}OIS_{it}^{2}+\beta_{7}OR_{it}\\ \;+\beta_{8}OR_{it}\times OIS_{it}+\beta_{10}OR_{it}\times \beta_{11}OIS_{it}^{2}\\ \;+\beta_{12}OE_{it}+\beta_{13}OE_{it}\times \beta_{14}OIS_{it}+\beta_{15}OE_{it}\\ \;\times \beta_{16}OIS_{it}^{2}+\mu_{it} \end{array}$$

where *i* indicates the number of observations, the β parameters are the coefficients that can be estimated in the hierarchical regression model, and the μ parameter is the error term in each equation.

Considering the inefficiency and estimated bias of the ordinary least squares regression model, this study conducted the fixed models to test the direct and moderating effects (58, 59).

## Results

### Regression analysis

The correlation results of this study are presented in Table 2. Since this study involves moderating effects, following previous studies (5, 60, 61), we used hierarchical regression to test our hypotheses. The results of hypothesis testing are presented in Table 3.

**Table 2:** Correlation matrix.

**Variables**	**1**	**2**	**3**	**4**	**5**	**6**	**7**	**8**	**9**
1. Potential patient education	1.000								
2. Realized patient education	0.383	1.000							
3. Online information sharing	0.531	0.133	1.000						
4. Online reputation	0.794	0.248	0.456	1.000					
5. Offline expertise	0.367	0.119	0.174	0.200	1.000				
6. Online time	0.632	0.250	0.203	0.182	0.374	1.000			
7. Gift	0.645	0.218	0.373	0.795	0.199	0.137	1.000		
8. Vote	0.600	0.231	0.302	0.658	0.3889	0.246	0.731	1.000	
9. Thank-you	0.582	0.214	0.318	0.654	0.298	0.195	0.750	0.899	1.000

**Table 3:** Results of hierarchical regression.

**Potential patient education (PPE)**	**Model 1**	**Model 2**	**Model 3**	**Model 4**
	**PPE**	**PPE**	**PPE**	**PPE**
Online information sharing (OIS)	0.340^***^ (0.003)	0.059^***^ (0.005)	0.376^***^ (0.010)	0.100^***^ (0.007)
Online reputation (OR)		0.467^***^ (0.002)		0.467^***^ (0.002)
OIS × OR		0.026^***^ (0.001)		0.028^***^ (0.001)
Offline expertise (OE)			0.063^***^ (0.007)	0.102^***^ (0.005)
OIS × OE			−0.012^***^ (0.003)	−0.017^***^ (0.002)
Online time	1.076^***^ (0.005)	1.035^***^ (0.003)	1.062^***^ (0.005)	1.012^***^ (0.003)
Gift	0.440^***^ (0.003)	0.0175^***^ (0.003)	0.444^***^ (0.003)	0.022^***^ (0.003)
Vote	0.104^***^ (0.006)	−0.013^***^ (0.004)	0.085^***^ (0.006)	−0.044^***^ (0.004)
Thank-you	0.055^***^ (0.007)	0.052^***^ (0.005)	0.062^***^ (0.007)	0.064^***^ (0.005)
Constant	2.152^***^ (0.030)	1.546^***^ (0.022)	2.091^***^ (0.033)	1.469^***^ (0.023)
*R* ^2^	0.773	0.893	0.773	0.895
**Realized patient education (RPE)**	**Model 5**	**Model 6**	**Model 7**	**Model 8**
	**RPE**	**RPE**	**RPE**	**RPE**
Online information sharing (OIS)	0.187^***^ (0.010)	0.151^***^ (0.024)	0.410^***^ (0.033)	0.357^***^ (0.036)
Online information sharing squire (OISS)	−0.041^***^ (0.002)	−0.063^***^ (0.007)	−0.071^***^ (0.008)	−0.087^***^ (0.009)
Online reputation (OR)		0.061^***^ (0.005)		0.055^***^ (0.005)
OIS × OR		0.011^**^ (0.004)		0.017^***^ (0.004)
OISS × OR		0.002 (0.001)		0.001 (0.001)
Offline expertise (OE)			0.050^***^ (0.010)	0.062^***^ (0.010)
OIS × OE			−0.077^***^ (0.010)	−0.080^***^ (0.011)
OISS × OE			0.011^***^ (0.002)	0.010^***^ (0.003)
Online time	0.299^***^ (0.006)	0.289^***^ (0.006)	0.308^***^ (0.006)	0.296^***^ (0.006)
Gift	0.086^***^ (0.004)	0.012^*^ (0.005)	0.086^***^ (0.004)	0.012^*^ (0.005)
Vote	0.074^***^ (0.008)	0.059^***^ (0.008)	0.080^***^ (0.008)	0.064^***^ (0.008)
Thank-you	0.007 (0.009)	0.000 (0.009)	0.008 (0.009)	0.001 (0.009)
Constant	5.165^***^ (0.040)	5.134^***^ (0.044)	4.945^***^ (0.047)	4.924^***^ (0.049)
*R* ^2^	0.106	0.114	0.108	0.116

* p < 0.050,

** p < 0.010,

*** p < 0.001 (2-tailed test).

H1 proposed the positive relationship between physicians' online information sharing (OIS) and potential patient education (PPE). The results in Model 1 indicate that OIS (β = 0.340, *p* < 0.001) is positive and significantly related to PPE. Thus, H1 is supported. As for the control variables, the coefficients of all are significant and positive (Online time, β = 1.076, *p* < 0.001; Gift, β = 0.440, *p* < 0.001; Vote, β = 0.104, *p* < 0.001; Thank-you, β = 0.055, *p* < 0.001).

**Figure 2:**
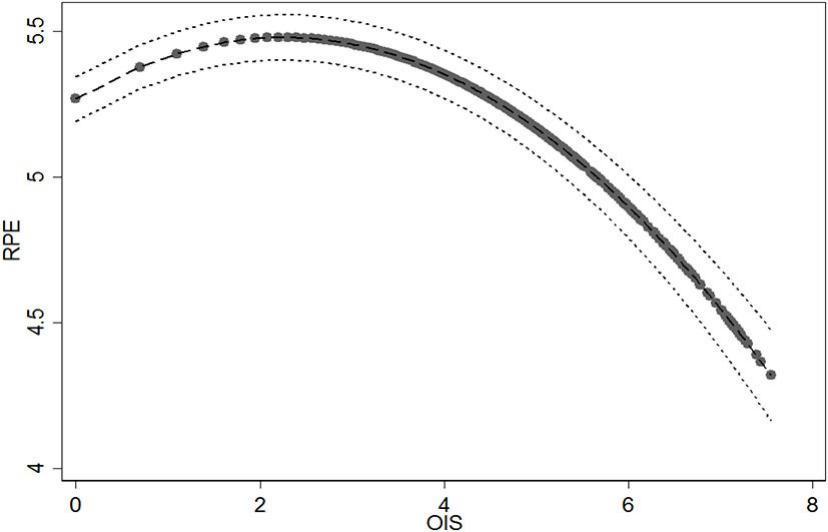
The relationship between online information sharing (OIS) and realized patient education (RPE).

H2 proposed the inverted U-shape relationship between online information sharing (OIS) and realized patient education (RPE). To test the hypothesized inverted U-shape relationship, we followed the suggestions of Haans et al. (62) to evaluate the coefficient estimates, slope significance, and turning point against data range. First, the results in Model 5 show that the coefficient of OIS is positive and significant (β = 0.187, *p* < 0.001), whereas the squared term of online information sharing (OISS) is negative and significant (β = −0.041, *p* < 0.001). We then tested the slopes of the OIS effect at both the low and the high ends of OIS. The slopes at the low (OIS=0; β = 0.187, *p* < 0.001) and the high (OIS=7.550; β = −0.438, *p* < 0.001) ends are both sufficiently steep and statistically significant. Third, the turning point of the curvilinear effect is calculated at OIS = 2.257, with a 95% confidence interval from 2.162 to 2.351, which is well within the data range. We also plotted the relationship between OIS and RPE (see Figure 2). Altogether, these results satisfy the inverted U-shape testing criteria of Haans et al. (62), rendering support for our H2. In addition, the effects of control variables are positive (Online time, β = 0.299, *p* < 0.001; Gift, β = 0.086, *p* < 0.001; Vote, β = 0.074, *p* < 0.001) and significant except for Thank-you.

**Figure 3:**
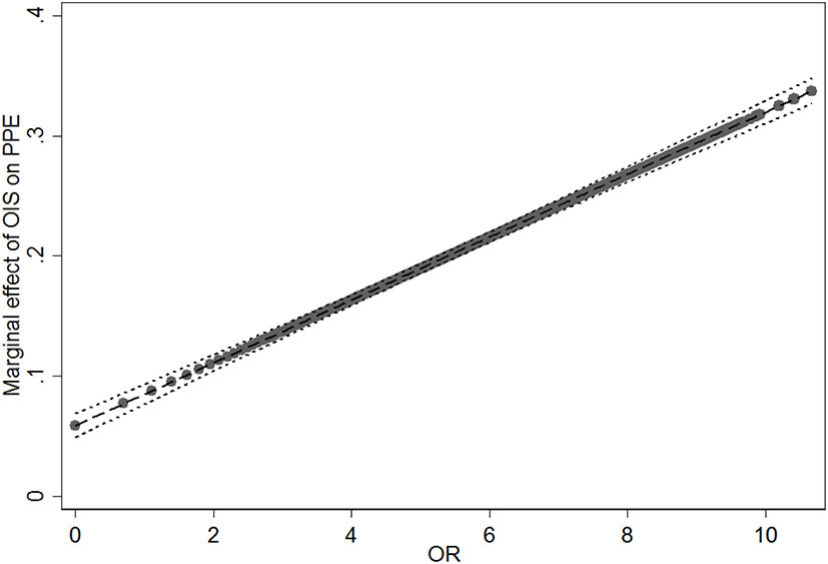
Moderating effect of online reputation (OR) on the relationship between online information sharing (OIS) and potential patient education (PPE).

H3 and H4 argued the moderating effect of physicians' online reputation (OR). In Model 2, the positive moderating effect of OR on the positive relationship between OIS and PPE is examined. The coefficient of the interaction term (OIS × OR) is significant and positive (β = 0.026, *p* < 0.001). Following Meyer et al. (63), we plotted the marginal effect of physicians' information sharing on potential patient education at different levels of physicians' online reputation (Figure 3). The results show that as the values of online reputation increase from 0 to 10.672, the slope of the relationship between physicians' information sharing and potential patient education becomes steeper. Thus, H3 is supported. Meanwhile, the negative moderating effect of physicians' OR on the inverted U-shape relationship between OIS and RPE is tested in Model 6. The relationship between the interaction of the squared term (OISS × OR) is not significant (β = 0.002, *p* > 0.050). Thus, H4 is not supported.

**Figure 4:**
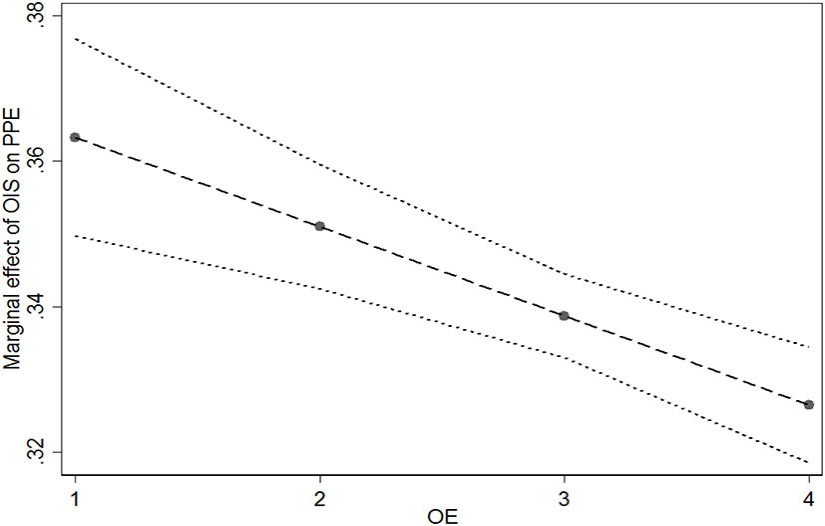
Moderating effect of offline expertise (OE) on the relationship between online information sharing (OIS) and potential patient education (PPE).

**Figure 5:**
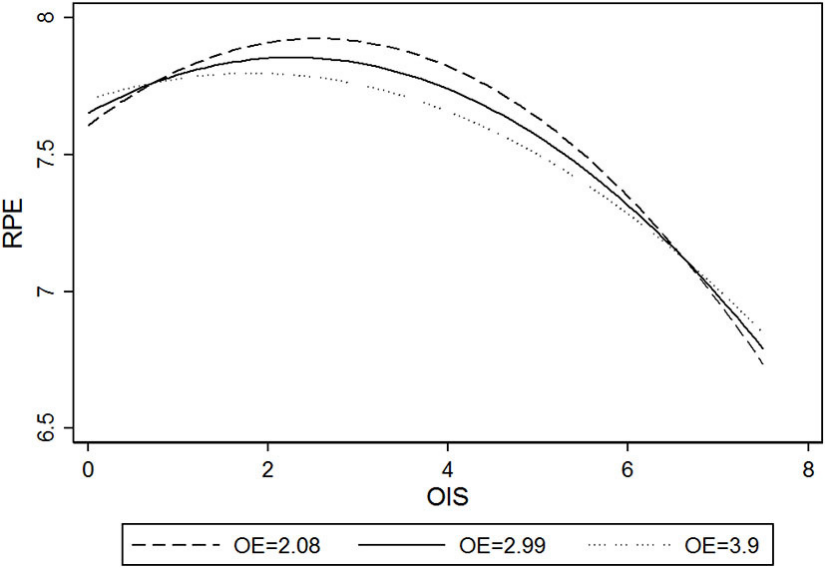
Moderating effects of offline expertise (OE) on the relationship between online information sharing (OIS) and realized patient education (RPE).

H5 and H6 posit the moderating effect of physicians' offline expertise (*OE*). H5 is tested in Model 3, the results show that the coefficient of the interaction term (*OIS*×*OE*) is negative and significant (β = −0.012, *p* < 0.001). Figure 4 illustrates the marginal effect of physicians' information sharing on potential patient education at different levels of physicians' offline expertise. The moderating effect shows that as the value of offline expertise increases from 1 to 4, the slope of the relationship between physicians' information sharing and potential patient education becomes flatter. Therefore, H5 is supported. As for the testing of H6, the results are presented in Model 7. The coefficient of the interaction term (*OIS*×*OE*) is negative and significant (β = −0.077, *p* < 0.001). Meanwhile, the coefficient of interaction of the squared term (*OISS*×*OE*) is positive and significant (β = 0.011, *p* < 0.001). Figure 5 shows the moderating effects of offline expertise (*OE*) on the relationship between online information sharing (*OIS*) and realized patient education (*RPE*). With the increase of *OE*, the inverted U-shape curve relationship between *OIS* and *RPE* becomes significantly flatter. Under the impact of physicians' offline expertise, realized patient education is less affected by physicians' information sharing. Thus, H6 is supported.

### Supplementary analysis

To test the robustness of our findings, following the suggestions of Guo et al. (64) and Wang et al. (65), this study applied full models to further test our moderating effects in Table 3. In terms of potential patient education, all moderators and interaction terms were entered according to Model 1, and the results are presented in Model 4. The coefficients of the interaction terms are significant (*OIS*×*OR*, β = 0.028, *p* < 0.001; *OIS*×*OE*, β = −0.017, *p* < 0.001). The results are consistent with Model 2 and Model 3, and H3 and H5 are further supported. In terms of realized patient education, all moderators and interaction terms were entered according to Model 5, and the results are presented in Model 8. The coefficient of the squared term about online reputation is insignificant (β = 0.001, *p* > 0.05), while that about offline expertise is significant (β = 0.010, *p* < 0.001). The results are consistent with Model 6 and Model 7, thereby further rejecting H4 and supporting H6.

We further conducted random effects regression models as supplementary analysis following previous studies (66, 67). The results are demonstrated in Table 4. Model 9 shows that the relationship between *OIS* and *PPE* is positive and significant (β = 0.340, *p* < 0.001), supporting H1. In Model 13, the coefficient of *OIS* is positive and significant (β = 0.186, *p* < 0.001), whereas the squared term (*OISS*) is negative and significant (β = −0.041, *p* < 0.001). Thus, H2 is also supported. Model 10 and Model 11 confirm the moderating effects of *OR* (β = 0.026, *p* < 0.001) and *OE* (β = −0.012, *p* < 0.001) on the relationship between *OIS* and *PPE*, and Model 12 also confirms the abovementioned relationship (*OIS*×*OR*, β = 0.028, *p* < 0.001; *OIS*×*OE*, β = −0.017, *p* < 0.001). The results provide evidence for supporting H3 and H5. Similar to the main analysis, the coefficient of the interaction of the squared term (*OISS*×*OR*) is not significant (β = 0.002, *p* > 0.001) in Model 14. Model 15 reports that the coefficient of the interaction term (*OIS*×*OE*) is negative and significant (β = −0.077, *p* < 0.001) and the coefficient of the interaction of the squared term (*OISS*×*OE*) is positive and significant (β = 0.010, *p* < 0.001). Model 16 also indicates that the interaction of the squared term *OISS*×*OR* is not significant (β = 0.001, *p* > 0.050), but the interaction of the squared term *OISS*×*OE* is significant (β = 0.010, *p* < 0.001). Thus, H4 is rejected and H6 is supported. In summary, the results are similar to the fixed effects and our results are robust.

**Table 4:** Results of the robust test.

**Potential patient education (PPE)**	**Model 9**	**Model 10**	**Model 11**	**Model 12**
	**PPE**	**PPE**	**PPE**	**PPE**
Online information sharing (OIS)	0.340^***^ (0.003)	0.059^***^ (0.005)	0.376^***^ (0.010)	0.100^***^ (0.007)
Online reputation (OR)		0.467^***^ (0.002)		0.467^***^ (0.002)
OIS × OR		0.026^***^ (0.001)		0.028^***^ (0.001)
Offline expertise (OE)			0.063^***^ (0.007)	0.102^***^ (0.005)
OIS × OE			−0.012^***^ (0.003)	−0.017^***^ (0.002)
Online time	1.076^***^ (0.004)	1.036^***^ (0.003)	1.063^***^ (0.005)	1.012^***^ (0.003)
Gift	0.439^***^ (0.003)	0.017^***^ (0.003)	0.443^***^ (0.003)	0.022^***^ (0.003)
Vote	0.103^***^ (0.006)	−0.014^***^ (0.004)	0.085^***^ (0.006)	−0.045^***^ (0.004)
Thank-you	0.054^***^ (0.007)	0.052^***^ (0.005)	0.062^***^ (0.007)	0.064^***^ (0.005)
Constant	2.148^***^ (0.030)	1.543^***^ (0.022)	2.087^***^ (0.033)	1.466^***^ (0.023)
*R* ^2^	0.773	0.893	0.773	0.895
**Realized patient education (RPE)**	**Model 13**	**Model 14**	**Model 15**	**Model 16**
	**RPE**	**RPE**	**RPE**	**RPE**
Online information sharing (OIS)	0.186^***^ (0.010)	0.150^***^ (0.024)	0.409^***^ (0.033)	0.355^***^ (0.036)
Online information sharing squire (OISS)	−0.041^***^ (0.002)	−0.063^***^ (0.007)	−0.070^***^ (0.008)	−0.087^***^ (0.009)
Online reputation (OR)		0.061^***^ (0.005)		0.055^***^ (0.005)
OIS × OR		0.012^**^ (0.004)		0.017^***^ (0.004)
OISS × OR		0.002 (0.001)		0.001 (0.001)
Offline expertise (OE)			0.050^***^ (0.010)	0.062^***^ (0.010)
OIS × OE			−0.077^***^ (0.010)	−0.080^***^ (0.010)
OISS × OE			0.010^***^ (0.002)	0.010^***^ (0.003)
Online time	0.299^***^ (0.006)	0.289^***^ (0.006)	0.308^***^ (0.006)	0.296^***^ (0.006)
Gift	0.086^***^ (0.004)	0.012^*^ (0.005)	0.086^***^ (0.004)	0.012^*^ (0.005)
Vote	0.075^***^ (0.008)	0.060^***^ (0.008)	0.081^***^ (0.008)	0.064^***^ (0.008)
Thank-you	0.006 (0.009)	−0.001 (0.009)	0.007 (0.009)	0.000 (0.009)
Constant	5.162^***^ (0.040)	5.131^***^ (0.044)	4.942^***^ (0.047)	4.922^***^ (0.049)
*R* ^2^	0.106	0.114	0.108	0.116

* p < 0.050,

** p < 0.010,

*** p < 0.001 (2-tailed test).

## Discussion

### Key findings

This study analyzed how physician online information sharing affects patient education by considering the contingent effects of physicians' online reputation as well as physicians' offline expertise. Based on a 6-month panel data of 61,566 physician-month observations collected from an OHP in China, this study generated three significant findings.

First, support for the attention hypotheses was found. Physicians' information-sharing behaviors are positively related to the potential patient education. As we know, patients are more likely to acquire valuable information for them when doctors post more medical or treatment instructions in OHCs (68, 69). Patients' attention may be drawn by themes of interest in health articles and patient visits will grow as physician information exchange increases (14). Also, an inverted-U shape curvilinear relationship exists between physician online information sharing and realized patient education. Patient has limited attention (37, 70), although at first they read online information carefully. As a process of goal-driven when patients read physician's online articles, information overload makes patients less interested in the articles written by doctors (24).

Second, this study verified the moderating effect of physician online reputation. Physician online reputation strengthens the effect of health information sharing on potential patient education. Reputation serves as an intangible asset for physicians, which reflects their popularity on the online health platform (71). In such cases, patients seeking information to understand a diagnosis will be attracted by that popular health information. Previous studies have also confirmed that patients prefer to trust doctors who have a higher medical quality and service attitude (71). Unfortunately, the hypothesis that physicians' online reputation steepens the inverted U-shape relationship between physician online information sharing and realized patient education is not supported. One possible explanation is that patients tend to read articles that could improve their health behaviors and health status regardless of the physicians' online reputation (72). Thus, the relationship between physician online information sharing and realized patient education is almost not influenced by physicians' online reputation.

Finally, the moderating effect of physician offline expertise was also identified in this study. Physician offline expertise weakens physician online information sharing and patients' visit. As we know, higher-level professionals have a larger chance of attracting patients' engaged, goal-driven attention (29). The number of articles they published has less of an impact on their page visits growth. Also, the curvilinear effect of physician online information sharing and realized patient education is flattened by physician offline expertise. This reveals that the trade-off between benefit and cost of attention determines patients whether or not to read (7). Information sharing from experts (physicians with high professional titles) is considered more valuable guidance (17); in this context, patient will visit the experts' personal page regardless of few paper publications or a large amount of shared information.

### Theoretical contributions

This study provides three theoretical contributions to the current literature. First, the study extends the attention literature by introducing the attention theory to track the mechanism of physician online knowledge sharing and patient education. Although the attention theory has been widely explored in the fields of user behaviors and information networks (24, 40), this theory is rarely used in OHPs to explore online patient education. As far as we know, we are among the first to apply attention theory to track the mechanism of physician online knowledge sharing and patient education on OHPs. It is worthwhile to emphasize that physician online information sharing and potential patient education is a linear relationship, while there is an inverted U-shape between online information sharing and realized patient education. According to attention theory, patients' visiting is similar to a stimulus-driven attention process, and the information sharing by physicians may stimulate potential patient education as patients' attention could be captured by the contents of interest in shared health articles (12, 23). However, realized patient education (patients' reading) is a goal-driven process where patients' information decision is the trade-off between the benefits and the costs of attention. As a result, information overload reduces patients' interest in reading articles by doctors (24). This study applies attention theory to uncover the different effects of physician online knowledge sharing on potential and realized patient education, which makes contributions to physician online knowledge sharing and patient education.

Second, this study extends the online reputation and patient education literature by uncovering the contingent effect of online reputation in the process of physicians' online information sharing. The physician online information sharing effect on patient education is a decision-making process depending on context (14, 30). However, few studies have explored how physicians' online information sharing affected patient education, considering the context (73, 74). Our study considers the contingent effects of physicians' online reputation and finds that physicians' online reputation positively moderates the effect of physicians' online knowledge sharing on patients' potential education. A high online reputation elevates the information supplied by the physician and attracts patients as a valuable and uncommon resource (53) in this context, physician online knowledge sharing is more likely to attract patient visiting for potential education. Therefore, our discoveries contribute to the studies of online information sharing and patient education.

Finally, this study enriches the offline expertise and patient education literature by uncovering the contingent effect of offline expertise in the process of physicians' online information sharing. The information decision-making process is the trade-off between the benefits and the costs of limited attention, and such behaviors are not independent of the context (14, 30). During the influence of stimulus-driven and goal-driven attention (22), offline expertise will increase the value of the information and raise the anticipated attention cost of reading health articles (29). Thus, we find that physician online expertise weakens the positive effect of physician online information sharing and potential patient education and flattens the curvilinear effect of physician online information sharing and realized patient education. In other words, this study reveals the moderating effects of offline expertise on potential and realized patient education, thereby contributing to the literature on offline expertise and patient education.

### Practical implications

This study has several practical implications for patients and physicians, as well as platform managers. First, patients should visit physicians' homepages and read physicians' articles to improve their health education. As we know, physician online information sharing provides information support, suggestions, and guidance to patients (5), which is important to potential patient education and realized patient education. To effectively conduct health management and improve medical knowledge, for instance, patients can visit doctors' homepages and read some recent or most accessed medical articles.

Second, physicians should rationally engage in online information sharing by publishing health articles. According to our findings, physician online information sharing positively affects potential patient education, while it has an inverted U-shape relationship with realized patient education. In the early stages, it is necessary for physicians to publish more health articles to attract patient visiting and reading, which is beneficial to improve potential and realized patient education. As the published articles increase to a certain level, physicians need to control the quantity of published health articles and improve the quality of articles to better educate patients. For example, when physician's volume of articles reaches a high level, they can focus on publishing high quality and attractive medical papers.

Finally, platform managers should provide physicians with guidance about online information sharing to improve patient education (75, 76). The results of this study show that physicians' online reputation intensifies the positive relationship between physician online information sharing and potential patient education. Platform managers should encourage physicians with high online reputations to publish more papers or articles to better educate patients. In addition, physicians' offline expertise hinders the positive relationship between online information sharing and potential patient education and flattens the inverted relationship between online information sharing and realized patient education. Thus, platform managers may encourage young or junior doctors (e.g., rewarding or monetary incentives) to share multiple articles in the early stages to strengthen potential and realized patient education at the same time.

### Limitations and future research

Although this research has produced interesting findings and contributed to both theory and practice, there are still some limitations that reveal where future research is needed. First, the findings of this research are based on data from the Chinese context, which may restrict the applicability to other nations. Second, this study only adopted physician online reputation and physician online expertise as moderators; other contexts that could be taken into account in the analysis of physicians' online knowledge sharing are neglected, such as information uncertainty (42). Future study may consider the context to further track our study. Finally, this study does not include mediators. In fact, the readability (e.g., exceeding patients' average reading level) of online resources may affect education of patients (77). Future research could introduce readability as a mediator to instigate online knowledge sharing and patient education.

## Conclusion

This study shed light on patient education on OHPs from the attention perspective. The results indicate that physician online information sharing influences potential patient education and realized patient education in different patterns due to the differences between patients' attention mechanisms in visiting pages and reading articles on OHPs. Moreover, physicians' online reputation and offline expertise play important contingent roles in the above process. Therefore, to improve patient education and public health management, proper guidance for physicians about rational engagement in online information sharing should be provided.

## Data availability statement

The original contributions presented in the study are included in the article/supplementary material, further inquiries can be directed to the corresponding author/s.

## Author contributions

All authors listed have made a substantial, direct, and intellectual contribution to the work and approved it for publication.

## Funding

This research was funded by National Natural Science Foundation of China (71902135), General program of Humanities and Social Sciences Research of the Ministry of Education in China (21YJA630129), General program of Natural Science Foundation of Guangdong Province (2022A1515011025), and Peiyang Scholar Foundation (2020XRG-0074).

## Conflict of interest

The authors declare that the research was conducted in the absence of any commercial or financial relationships that could be construed as a potential conflict of interest.

## Publisher's note

All claims expressed in this article are solely those of the authors and do not necessarily represent those of their affiliated organizations, or those of the publisher, the editors and the reviewers. Any product that may be evaluated in this article, or claim that may be made by its manufacturer, is not guaranteed or endorsed by the publisher.
